# Aspirin prevents colorectal cancer metastasis in mice by splitting the crosstalk between platelets and tumor cells

**DOI:** 10.18632/oncotarget.8655

**Published:** 2016-04-08

**Authors:** Paloma Guillem-Llobat, Melania Dovizio, Annalisa Bruno, Emanuela Ricciotti, Valerio Cufino, Angela Sacco, Rosalia Grande, Sara Alberti, Vincenzo Arena, Mariangela Cirillo, Carlo Patrono, Garret A. FitzGerald, Dieter Steinhilber, Alessandro Sgambato, Paola Patrignani

**Affiliations:** ^1^ Department of Neuroscience, Imaging and Clinical Science, Section of Cardiovascular and Pharmacological Sciences, and CeSI-MeT, “G. d'Annunzio” University, School of Medicine, Chieti, Italy; ^2^ Institute for Translational Medicine and Therapeutics, University of Pennsylvania, School of Medicine, Philadelphia, PA, USA; ^3^ Institute of General Pathology, Catholic University School of Medicine, Rome, Italy; ^4^ Institute of Pathologic Anatomy, Catholic University, School of Medicine, Rome, Italy; ^5^ Institute of Pharmacology, Catholic University, School of Medicine, Rome, Italy; ^6^ Institute of Pharmaceutical Chemistry, Goethe University, Frankfurt, Germany

**Keywords:** aspirin, colorectal cancer, metastasis, platelets, epithelial-mesenchymal transition

## Abstract

We investigated whether platelets prime colon cancer cells for metastasis and whether pharmacological inhibition of platelet function may prevent it. Coculturing HT29 human colon carcinoma cells with human platelets led to the induction of mesenchymal-like cancer cells characterized by downregulation of E-cadherin and upregulation of Twist1, enhanced cell mobility and a proaggregatory action on platelets. These changes were prevented by different antiplatelet agents, aspirin[an inhibitor of cyclooxygenase(COX)-1], DG-041[an antagonist of prostaglandin(PG)E_2_ EP3 receptor] and ticagrelor (a P2Y12 receptor antagonist). The injection of HT29 cells, exposed to platelets *in vitro*, into the tail vein of humanized immunodeficient mice led to higher incidence of lung metastasis compared to the injection of untreated HT29 cells. This effect was associated with enhanced systemic biosynthesis of thromboxane(TX)A_2_ and PGE_2_ *in vivo*. Platelet COX-1 inhibition by aspirin administration to mice prevented the increased rate of metastasis as well as the enhanced production of TXA_2_ and PGE_2_ induced by the *in vitro* priming of HT29 cells by platelets. In conclusion, targeting platelet COX-1 with low-dose aspirin exerts an antimetastatic action by averting the stem cell mimicry of cancer cells associated with enhanced proaggregatory effects induced by platelet-tumor cell interactions. These effects may be shared by other antiplatelet drugs.

## INTRODUCTION

The acquisition of a mesenchymal-like phenotype by colorectal cancer cells has been recognized as a relevant phenomenon in the development of metastasis by promoting cancer cell intravasation and extravasation [1]. This process involves the switch of carcinoma cells from an epithelial to a mesenchymal-like phenotype, via a process designated epithelial-to-mesenchymal transition (EMT) which is triggered by autocrine and/or paracrine factors [1,2].

A feed-forward loop is operative between platelets and tumor cells: tumors can stimulate platelet activation and activated platelets can, in turn, promote tumor growth and metastasis [3]. It has been recently shown that EMT can be induced by the interaction of platelets with tumor cells [4,5]. However, platelets also mediate other processes which contribute to cancer metastases, including (i) the formation of platelet aggregates surrounding tumor cells that might support tumor cell survival and protection from immune elimination and (ii) the promotion of the adhesion of tumor cells to the endothelium thus leading to tumor cell arrest and extravasation [6].

Post-hoc analyses of randomized controlled trials with daily aspirin, designed to evaluate its efficacy in cardiovascular prevention, have shown that aspirin, at doses as low as 75-100 mg/day, reduces the incidence of cancer deaths possibly as a consequence of the prevention of distant metastases [7,8]. This chemopreventive effect was also seen in the Thrombosis Prevention Trial (TPT) [9] with a controlled-release matrix formulation of aspirin 75 mg daily which restricts the inhibitory effect of aspirin on platelet cyclooxygenase (COX)-1 to the presystemic compartment, preserving the increase in vascular prostacyclin (PGI_2_) evoked by systemic administration of bradykinin, as reflected by the urinary levels of 2,3-dinor-6-keto-PGF_1__α_(PGI-M), a major metabolite of vascular PGI_2_ [10]. This finding suggests that platelet activation is involved in the development of cancer metastases. However, the mechanism of action of aspirin in the prevention of cancer metastases is still under debate.

The induction of cancer cell EMT by platelets may be involved in the early steps of the metastatic process by allowing cancer cell mobilization into the circulation. Moreover, the mesenchymal-like phenotype of cancer cells may promote hematogenous dissemination through the expression of megakaryocyte gene products which activate platelets and the coagulation cascade [11].

We performed the present study to clarify the molecular mechanisms of action of aspirin in the prevention of hematogenous colorectal cancer (CRC) metastases. In particular, we aimed to address whether selective inhibition of platelet COX-1 activity by aspirin may prevent the development of a mesenchymal-like invasive phenotype in HT29 human colon carcinoma cells. Another objective of this study was to verify whether the mesenchymal-like invasive phenotypic switch induced in cancer cells by the cross-talk with platelets is characterized by enhanced prothrombotic potential and whether this event is sensitive to aspirin. Finally, we explored whether aspirin prevention of platelet-induced EMT and migration properties of cancer cells is a common mechanism shared by other antiplatelet agents with different mechanisms of action, such as ticagrelor, an antagonist of the platelet P2Y12 receptor for adenosine diphosphate (ADP) [12,13], and DG-041, an antagonist of the platelet EP3 receptor for prostaglandin (PG)E_2_ [14].

Our results suggest that low-dose aspirin and possibly other antiplatelet agents may represent effective antimetastatic agents by averting the stem cell mimicry of cancer cells and their proaggregatory properties on platelets, thus inhibiting both the early steps of the cancer metastatic process and its progression via hematogenous dissemination to multiple organs.

## RESULTS

### The administration of low-dose aspirin constrains enhanced metastatic potential of colon cancer cells induced by platelets

We have previously shown that human platelets prime human HT29 colon carcinoma cells *in vitro* to acquire a gene expression profile characteristic of a more malignant phenotype [5]. Here, we investigated whether the exposure of HT29 cells to human platelets *in vitro* enhances their ability to form lung metastases *in vivo*. We developed an experimental mouse model of hematogenous metastases, where immunodeficient NOD-*scid IL2Rγ^null^* (NSG) mice were injected via the tail vein with HT29 cells and the formation of lung metastases was quantified after 7 days. We used NSG mice because they allow studying the role of platelet activation in the metastatic process without the influence of the innate immune response. Moreover, it represents a fast model of human cancer lung metastases. The time-point of one week was selected to end the experiments since in preliminary feasibility studies we found that at later time points HT29 control cells induced a total tumor replacement in both lungs.

**Figure 1:**
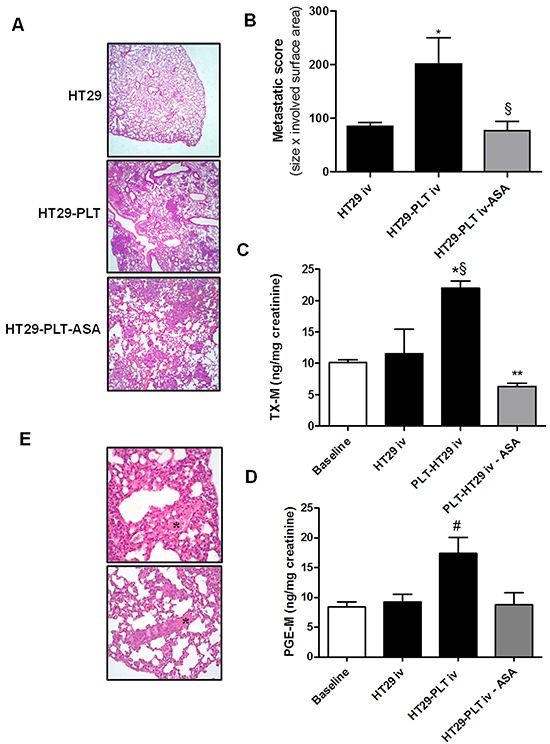
The administration of low-dose aspirin constrains enhanced metastatic potential of mesenchymal-like cancer cells induced by platelets **A.** and **B.** HT29 cells (1×10^6^) were cultured alone (HT29) or cocultured with platelets (1×10^8^) (HT29-PLT) for 40h; after the incubation, HT29 cells were extensively washed with PBS to remove platelets, harvested with trypsin, resuspended in HBSS (at a concentration of 5×10^6^ cells/mL); 200 μL of cell suspension (corresponding to 1×10^6^ cells) were injected into the lateral tail vein of NSG mice (n=5 each group). In HT29-PLT-ASA group (n=5), mice were treated with aspirin (20 mg/kg, p.o., once a day) starting from 4 days before the injection of HT29 cells cocultured with platelets and up to 7 days after the injection of the cells; one week from the injection, mice were sacrificed, lungs were collected, formalin-fixed and submitted for histopathology and the hematoxylin-eosin (H&E) stained microscopic sections were analyzed for metastatic score (obtained by combining the size of detected lesions × the surface area involved); mean ± SEM (n=5), *P<0.05 vs HT29 and ^§^P<0.05 vs HT29-PLT. **C.** and **D.** Twenty four-h urine samples were collected to assess the urinary excretion of TX-M and PGE-M; mean ± SEM (n=5), *P<0.05 vs HT29, §P<0.01 vs baseline. **P<0.01 vs HT29-PLT, #P<0.05 vs all the other conditions. **E.** H&E stain showing fibrin and red blood cells in lung sections. (*) In the bottom panel a thrombus containing aggregates of neoplastic cells is shown. Original magnification 20x and 40x.

Formalin-fixed, paraffin-embedded lungs were sectioned and stained with hematoxylin-eosin and Figure 1A shows examples of the microscopic fields that we scored. Histopathologic analysis revealed the presence of well-established micrometastases diffusely disseminated within both lungs at this time-point. The metastatic score (obtained by combining the size of detected lesions × the surface area involved) in the lungs of mice inoculated with HT29 cells cultured alone displayed and average value of 2.6±0.4.

To investigate the influence of platelets on the metastatic potential of colon cancer cells, HT29 cells were exposed to human platelets *in vitro* for 40h, then platelets were washed away and tumor cells (substantially devoid of any platelets, Supplementary Figure S1) were injected into the tail vein of mice. As shown in Figure 1B, the exposure of HT29 cells to platelets *in vitro* caused a significant increase in the development of metastases. One of the mice in the platelet-treated HT29 group displayed a complete tumor replacement in some sections (Figure 1A, middle panel and data not shown).

In order to verify whether the injection of HT29 cells was associated with enhanced platelet activation *in vivo* we assessed the urinary levels of TX-M which is a major enzymatic metabolite of TXA_2_, a potent stimulus for platelet activation. TX-M is an index of the systemic biosynthesis of TXA_2_ mainly derived from platelets [15]. As shown in Figure 1C, the i.v. administration of HT29 cells did not significantly alter urinary TX-M level versus baseline values (10.10 ±0.4ng/mg creatinine). In contrast, urinary TX-M levels were significantly enhanced in mice injected with HT29 cells exposed *in vitro* to human platelets for 40h (Figure 1C).

This finding suggests that platelets may prime cancer cells to enhance their pro-thrombotic properties. Since PGE_2_ elicits a wide range of biological effects associated with cancer [16], we measured the urinary levels of PGE-M (a major enzymatic metabolite of PGE_2_, which is an index of the systemic biosynthesis of PGE_2_ *in vivo*) considered a potential biomarker of cancer risk and disease progression [17]. As shown in Figure 1D, PGE-M levels were significantly higher than those detected at baseline or in mice injected with HT29 cells not exposed to platelets *in vitro*.

Next we studied the effects of aspirin on metastasis formation *in vivo* by treating mice with a dose of the drug which preferentially inhibits platelet rather than extraplatelet sources of COX-dependent prostanoid biosynthesis. Aspirin 20mg/kg was administered daily by oral gavage to mice from 4 days before to a week after HT29 cell injection. This dose of aspirin corresponds to a human dose of 150 mg daily [18]. This dose of aspirin almost completely suppressed platelet COX-1 activity evaluated by assessing the production of TXB_2_ in clotting whole blood incubated at 37°C for 1h (an index of the maximal biosynthetic capacity of platelet COX-1 [19])(Supplementary Figure S2A). This effect was associated with a marginal, non-significant, inhibitory effect on the systemic biosynthesis of PGI_2_ *in vivo*, confirming the relative biochemical selectivity of this aspirin regimen (Supplementary Figure S2B).

The *in vivo* administration of aspirin prevented the increase of the metastatic potential of HT29 cells induced by *in vitro* exposure to platelets (Figure 1A and 1B). This effect of aspirin was associated with prevention of the increased systemic biosynthesis of TXA_2_ and PGE_2_ induced by the vascular injection of HT29 cells exposed to platelets *in vitro* (Figure 1C and 1D, respectively).

The administration of aspirin reduced both the basal rate of systemic biosynthesis of TXA_2_ and its induction by cancer cells (Figure 1C). In contrast, aspirin inhibited enhanced systemic PGE_2_ biosynthesis in mice injected with platelet-primed HT29 cells but did not affect baseline values of PGE-M (Figure 1D). Thus, the enhanced biosynthesis of TXA_2_ and PGE_2_ detected *in vivo* after the injection of platelet-primed cancer cells seems to be triggered by the interaction of human cancer cells with mouse platelets *in vivo*.

Altogether our findings show that platelets prime HT29 cells for metastases coincidently to the acquisition of an ability to trigger platelet TXA_2_ and PGE_2_ biosynthesis *in vivo*. Aspirin prevented platelet activation induced by cancer cells and this effect was associated with a reduction of pulmonary metastases.

Interestingly, besides metastatic nodules in lung tissue, we also observed the presence of tumor thrombi in pulmonary blood vessels of treated mice. Blood vessels appeared full of tumor emboli, which clumped together with platelets and resulted in the obstruction of the lumen. The presence of fibrin and red blood cells was also evident in most of them (Figure 1E and data not shown).

### Platelets induce EMT and promote migration of HT29 cells through an aspirin-sensitive mechanism

We aimed to clarify whether platelet-cancer cell cross-talk induces mesenchymal-like cancer cells *in vitro* and whether the selective inhibition of platelet COX-1 activity by aspirin may prevent this phenomenon. Thus, we performed cocultures of platelets with HT29 cells and assessed the expression levels of E-cadherin, a typical marker of the epithelial phenotype, and its transcriptional regulator Twist1. It has been reported that overexpression of Twist1, a basic helix-loop-helix transcription factor down-regulates E-cadherin expression [20,21].

**Figure 2:**
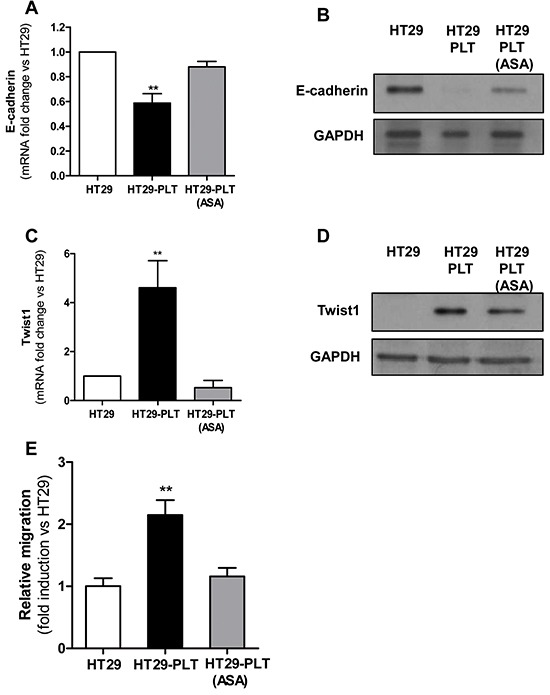
Platelets induce EMT in HT29 cells through an aspirin-sensitive mechanism Platelets were pre-treated for 30 min with vehicle (DMSO) or aspirin 300μM (to completely suppress platelet COX-1 activity). After an extensive washing (to remove vehicle or the drug), 1×10^8^ platelets were added to HT29 cells (1×10^6^) [HT29-PLT and HT29-PLT (ASA), respectively) for 20h (for gene expression analyses) or 40h (for protein expression analyses). As control condition, HT29 cells (1×10^6^) were cultured alone (HT29) for 20h or 40h. After the incubation, HT29 cells were extensively washed with PBS to remove platelets, harvested with trypsin. **A.** and **B.** E-cadherin and **C.** and **D.** Twist1 mRNA and protein levels (normalized to GAPDH) were assessed by qPCR and Western blot, respectively. Results are shown as relative expression (fold change) compared to HT29 cells cultured alone. Data are reported as mean ± SEM (n=4). **E.** HT29 cells (1×10^6^) were cultured alone (HT29) or with platelets (1×10^8^) pre-treated with aspirin (300μM)[HT29-PLT(ASA)] or vehicle (DMSO) (HT29-PLT) for 40h (as described above) and analysed for migratory properties using Boyden chamber. In brief, after the incubation, HT29 cells were detached by typsin, counted, resuspended in complete culture medium and seeded (1×10^5^ cells per insert) onto the upper chamber of transwell filters in 24-well multiplates. Cells were allowed to migrate for 40h, at 37°C in 5% CO_2_. Data are reported as fold induction vs HT29 cells cultured alone. Data are presented as mean ± SEM (n=4). **P <0.01 vs all the other conditions.

The interaction of platelets with HT29 cells was associated with a significant reduction in E-cadherin mRNA and protein levels (Figures 2A and 2B, respectively). Next, we explored whether platelets may influence Twist1 levels in HT29 cells. In cocultures of platelets and cancer cells, Twist1 was upregulated (both mRNA and protein) (Figures 2C and 2D, respectively).

Exposure of platelets to aspirin (to suppress COX-1-dependent prostanoid generation) and then washing the drug before the addition to HT29 cells, prevented the down-regulation of E-cadherin (Figure 2A and 2B) and upregulation of Twist1 (Figure 2C and 2D).

We also studied whether the EMT phenotype induced in HT29 cells by platelets was associated with enhanced cell motility, as assessed by the Boyden chamber assay. As shown in Figure 2E and Supplementary Figure S3A, platelets increased the migratory capacity of HT29 cells *in vitro*. In order to verify whether platelets act through an aspirin-sensitive mechanism, they were pretreated with aspirin and extensively washed before the addition to HT29 cells. As shown, in Figure 2E and Supplementary Figure S3A, aspirin pre-treatment prevented platelet-induced migration of HT29 cells.

### Aspirinated platelets fail to induce a prothrombotic phenotype in HT29 cancer cells

**Figure 3:**
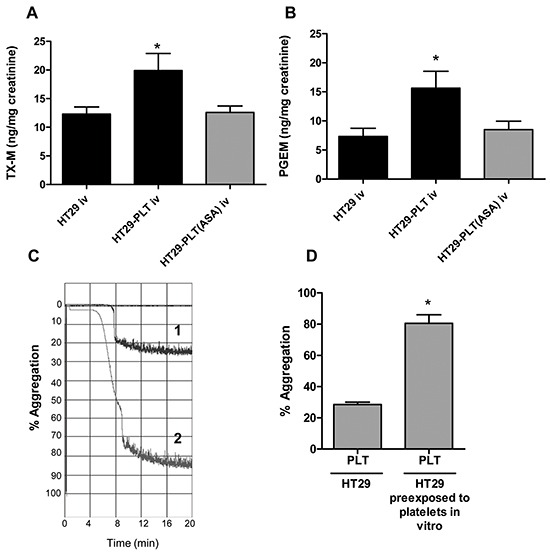
Aspirinated platelets fail to induce a prothrombotic phenotype in HT29 cancer cells **A.** and **B.** HT29 cells (1×10^6^) were cultured alone or cocultured with platelets (1×10^8^), pre-treated for 30min with vehicle (DMSO) (HT29-PLT) or aspirin 300μM (to completely suppress platelet COX-1 activity, HT29-PLT(ASA) for 40h (as described in the legend to Figure 2); after an extensive wash to remove platelets, HT29 cells were harvested with trypsin and resuspended in HBSS (at the concentration of 5×10^6^ cells/mL); 200 μl of cell suspension (corresponding to 1×10^6^ cells) were injected into the lateral tail vein of NSG mice. 24h urine samples were collected and urinary excretion of TX-M(A) and PGE-M(B) was measured. Data are reported as mean ± SEM (n=5).*P<0.05 vs HT29 and HT29-PLT(ASA). **C.** HT29-induced platelet aggregation was assessed in washed platelets isolated from fresh whole blood human samples and anticoagulated with a 1/6 volume of acid-citrate dextrose. Platelet-rich plasma (PRP) was obtained by blood centrifugation at 200g for 10min without brake. Platelets were sedimented by centrifugating PRP at 700g for 15min without brake and then they were resuspended in Hepes buffer pH 7.4 (10mM Hepes, 145mM NaCl, 5mM KCl, 0.5mM Na_2_HPO_4_ and 6mM glucose) at a concentration of 2×10^8^/mL. Two-hundred μl of platelets (0.4×10^8^ cells) were placed in the light aggregometer and incubated for 2min at 37°C after the addition of 1mM MgSO_4_ and 1mM CaCl_2_. After the incubation, platelets were subjected to stirring (900rpm) prior to the addition of tumor cells. TCIPA was initiated by the addition of 50μl of HT29 cells [cultured alone (trace 1) or cocultured with platelets (trace 2) for 40h *in vitro*, as described above], resuspended in Hepes buffer at the final concentration of 0.8×10^7^/mL, and the reaction was monitored for up to 20min. **D.** Platelet aggregation was expressed as % of the maximum aggregation rate, *P<0.05 vs HT29.

In the present study, we have shown that HT29 cells exposed *in vitro* to platelets acquire a mesenchymal-like invasive phenotype associated with enhanced capacity to activate platelets *in vivo* and that the exposure of HT29 cells to aspirinated platelets prevented the induction of EMT and migration in cancer cells. Next, we aimed to investigate whether platelets, pre-treated with aspirin *in vitro* and cocultured with HT29 cells for 40h, lose their capacity to induce a proaggregatory phenotype of cancer cells *in vivo*. As shown in Figure 3A and 3B, HT29 cells cultured with aspirin-treated platelets *in vitro* before the injection into the tail vein of NSG mice, did not enhance the systemic biosynthesis of TXB_2_ and PGE_2_ *in vivo*, respectively.

### Platelets prime HT29 cells to acquire a pro-aggregatory phenotype

We studied whether platelet-cancer cell interactions (associated with EMT induction in HT29 cells) could influence the capacity of HT29 cells to induce platelet aggregation (known as tumor-cell induction of platelet aggregation, TCIPA [3]). Thus, HT29 cells were cultured alone or with human platelets and after 40h, the cells were washed to remove the platelets (Supplementary Figure S1); then, HT29 cells cultured alone or cocultured with platelets were mixed with washed human platelets and platelet aggregation was measured by light aggregometry. As shown in Figure 3C and 3D, HT29 cells cultured alone showed a limited ability to induce platelet aggregation. Differently, HT29 cells cultured in the presence of platelets caused a complete platelet aggregation. These findings strongly support the hypothesis that HT29 cells undergoing EMT are characterized by enhanced ability to activate platelets.

### Prostanoids produced in platelet-HT29 cell cocultures are mainly derived from platelet COX-1

During the incubation of platelets with cancer cells, large amounts of TXB_2_ were released (109±38ng/mL) which were significantly higher than those detected in HT29 cells and platelets cultured alone (Supplementary Figure S2C). The pre-treatment of platelets with aspirin caused virtually complete suppression of TXB_2_ levels, consistent with the role of platelet COX-1 in its generation. However, HT29 cells do not express detectable levels of the TXA_2_ receptor TP (Supplementary Figure S4A and ref [5]), thus excluding a functional role of this prostanoid in the phenotypic changes induced by platelets in these cancer cells.

PGE_2_(1.11±0.18 ng/mL; 2-4.5nM) was also detectable in platelet-HT29 cell cocultures, though, at lower concentrations than TXB_2_ (Supplementary Figure S2D). These PGE_2_ levels were significantly higher than those detected in HT29 cells and platelets cultured alone (Supplementary Figure S2D). Similarly to TXB_2_, PGE_2_ was mainly derived from platelet COX-1 activity. In fact, exposure of platelets to aspirin caused a 90% reduction in PGE_2_ levels. Residual PGE_2_ levels were most likely derived from the HT29 cell COX-2 pathway, as previously reported [5]. Despite being produced at lower levels than TXA_2_, in light of HT29 cells expressing the PGE_2_ receptors subtypes EP1, EP2, and EP4 (Supplementary Figure S4A and ref [5]). Altogether these results are consistent with a role of platelet-derived PGE_2_ in the aspirin-sensitive mechanism responsible for the platelet-induced EMT.

### PGE_2_-induced EMT in HT29 cells

**Figure 4:**
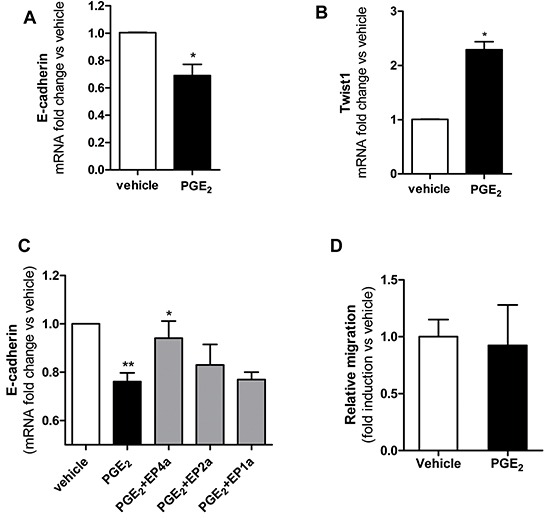
PGE_2_ induced EMT and migration of HT29 cells **A.** and **B.** mRNA levels of E-cadherin and Twist1 (normalized to GAPDH), respectively, were analysed by qPCR in HT29 cells (1×10^6^) treated with PGE_2_(5nM) or vehicle (DMSO) for 20h. Results are shown as relative expression (fold change) compared to vehicle. Data are reported as mean ± SEM (n=3). **C.** HT29 cells were incubated for 1h with EP antagonists: EP4a: L-161,982(150 nM); EP2a: PF 04418948 (160 nM); EP1a: SC 51322(120 nM); then PGE_2_(5 nM) was added to the culture for 20h and E-cadherin mRNA levels were assessed by qPCR, fold changes vs vehicle are shown. Mean ± SEM (n=3). **D.** HT29 cells were allowed to migrate in transwell filters (as described in the legend to Figure 2) in the presence of PGE_2_ (5nM) or vehicle and after 40h the fold changes of migrated cell number vs vehicle was assessed. Data are presented as mean ± SEM (n=5). (A, B) *P < 0.05 vs vehicle; (C)**P<0.01 vs vehicle, *P<0.05 vs PGE_2_.

In HT29 cells cultured alone, exogenous PGE_2_ (5nM a concentration which can be measured in platelet-HT29 cell coculture medium) caused down-regulation of E-cadherin mRNA associated with upregulation of Twist1 mRNA (Figures 4A and 4B, respectively). Using specific antagonists for the 3 EP receptors expressed in HT29 cells, i.e. SC51322 for EP1 [22], PF04418948 for EP2 [23] and L-161,982 for EP4 [24], we found that PGE_2_-dependent downregulation of E-cadherin in HT29 cells occurred through EP4 (Figure 4C).

At this concentration, PGE_2_ did not induce migration of HT29 cells, as assessed in the Boyden chamber (Figure 4D).

### Exogenous PGE_2_ abrogated the inhibitory effects of aspirinated platelets on EMT but not on cancer cell migration

**Figure 5:**
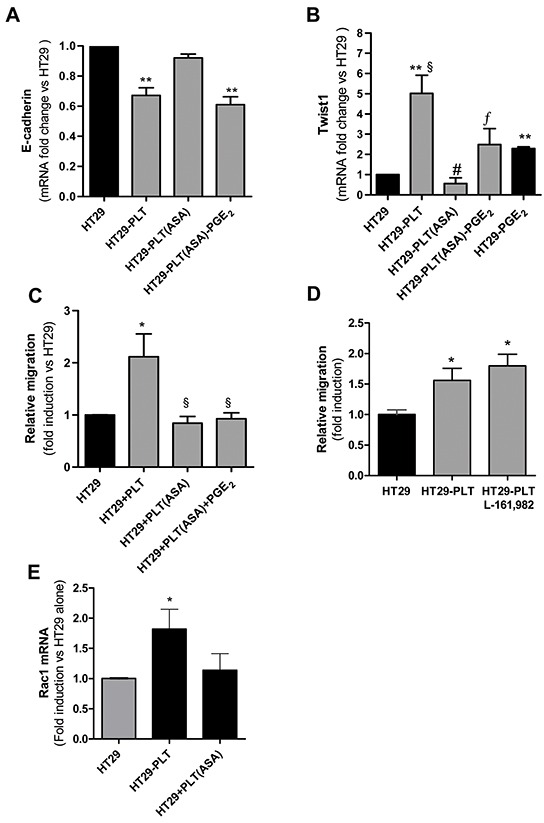
Effects of exogenous PGE_2_ on the inhibitory effect of aspirin on EMT and migration of HT29 cells exposed to platelets Platelets were pre-treated for 30min with vehicle (DMSO) or aspirin 300μM (to completely suppress platelet COX-1 activity); then, platelets were extensively washed (to eliminate vehicle or the drug) and 1×10^8^ cells were added to HT29 cells (1×10^6^) [HT29-PLT and HT29-PLT(ASA), respectively] for 20h. As control condition, HT29 cells (1×10^6^) were cultured alone for 20h (HT29). In some conditions, exogenous PGE2(5 nM) was added to HT29-PLT(ASA) for 20h. **A, B.** and **C.** HT29 cells were extensively washed with PBS to remove platelets, harvested with trypsin and assayed for mRNA of E-cadherin, Twist1 by qPCR and migratory property by the Boyden chamber assay (as reported in the legend to Figure 2), respectively. **D.** HT29 cells (1×10^6^) were cultured with platelets (1×10^8^) in the presence of vehicle (DMSO) or EP4 antagonist (L-161,982, 10 μM) for 40h; the migratory capacity was assessed by Boyden chamber, as described in the legend to Figure 2. **E.** In HT29 cells cultured alone (1×10^6^) or cocultured with platelets pre-treated with vehicle (DMSO) or aspirin (300μM), the fold induction of RAC1 mRNA was assessed by qPCR. Values are reported as mean ± SEM(n=4); (A)**P<0.01 vs HT29 alone and HT29-PLT(ASA); (B) **P<0.01 vs HT29 alone, §P<0.05 vs HT29-PGE2, #P<0.01 vs HT29-PLT, ¦P<0.05 vs HT-PLT(ASA); (C) *P<0.05 vs HT29, §P<0.05 vs HT29-PLT;(D) *P<0.05 vs HT29; (E) *P<0.05 vs HT29 and HT-PLT(ASA).

As shown in Figure 5A, exogenous PGE_2_(5nM, a concentration detectable in the coculture medium) rescued the inhibitory effect of aspirinated platelets on the changes of E-cadherin expression induced by the interaction of platelets with HT29 cells.

In contrast, the reduction of Twist1 expression by aspirinated platelets was only partially reversed (Figure 5B). In the same experiment, we compared the effect of PGE_2_ in HT29 cells cultured alone. PGE_2_ was less effective than platelets in up-regulating Twist1 (Figure 5B).

We also assessed the effects of exogenous PGE_2_ on the migratory properties of HT29 cells cocultured with aspirinated platelets. As shown in Figure 5C and Supplementary Figure S3B, PGE_2_ did not rescue the inhibition of HT29 cell migration by platelets pre-exposed to aspirin.

To confirm that endogenous PGE_2_ produced in the coculture of platelet and HT29 cells was not sufficient to induce cancer cell migratory properties, the effect of the EP4 antagonist L-161,982 [24] was evaluated. As shown in Figure 5D, L-161,982 (even at 10 μM) did not affect the migratory capacity of HT29 cells induced by the interaction with platelets.

These results suggest that: (i) the induction of Twist1 in HT29 cells by platelets involves the contribution of released PGE_2_ together with other signaling pathways derived from the cross-talk between platelets and cancer cells; (ii) both mechanisms are mitigated by the selective inhibition of platelet function by aspirin and (iii) PGE_2_ produced by platelet-cancer cell interactions takes part in EMT but not in the formation of a migratory cancer cell.

In addition to E-cadherin downregulation, Twist1 may contribute to the development of a motile mesenchymal-like cancer cell phenotype through the activation of RAC1 (Ras-related C3 botulinum toxin substrate 1), a small G-protein of the Rho family [25]. This prompted us to study the expression of RAC1 in platelet-HT29 cell cocultures. As shown in Figure 5E, RAC1 mRNA levels were induced by the interaction of cancer cells with platelets. Interestingly, platelets pre-exposed to aspirin showed a reduced capacity to induce RAC1 (Figure 5E).

### Antagonism of EP3 affects EMT and migration in platelet-cancer cell cocultures

EP3 receptors, undetectable in HT29 cells, are highly expressed in platelets (Supplementary Figure S4B) where they couple to a Gαi-type G protein and the activation opposes the increase in cAMP [26].

**Figure 6:**
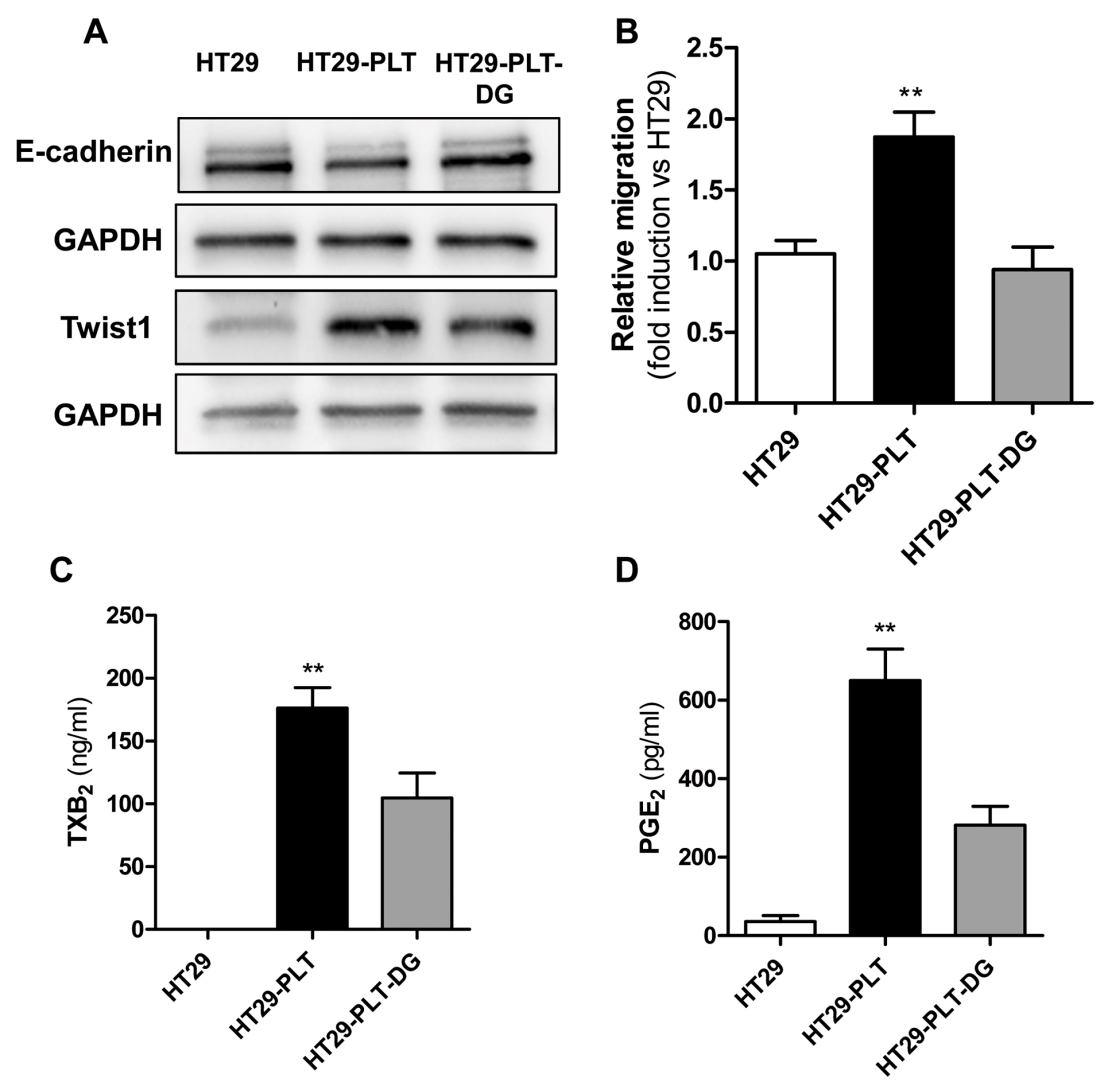
Blockage of EP3 affects EMT and migration in platelet-HT29 cell cocultures HT29 cells were cultured alone (1×10^6^, HT29) or cocultured with platelets (1×10^8^), in the absence (HT29-PLT) or presence of the EP3 antagonist DG-041 (3 μM) (HT29-PLT-DG) for 40h. HT29 cells were extensively washed with PBS to remove platelets, harvested with trypsin and the protein expression levels of E-cadherin and Twist1 (normalized to GAPDH) were assessed by Western blot (one of three representative Western blots is shown) **A.** and the migratory properties by using the Boyden chamber assay (as reported in the legend to Figure 2) **B.** **P<0.01 vs HT29 and HT29-PLT-DG. **C.** and **D.**TXB_2_ and PGE_2_ levels, respectively, were measured in culture media of HT29 cells either cultured alone (HT29), cocultured with platelets (HT29-PLT) in the absence and presence of DG-041 (HT29-PLT-DG) for 40 h. **P<0.01 vs HT29 and HT29-PLT-DG. Data are reported as mean ± SEM (n=3).

As shown in Figure 6A, DG-041, a potent and highly specific EP3 antagonist [14], partially prevented the down-regulation of E-cadherin and upregulation of Twist. Interestingly, the antagonist also prevented the enhanced migratory capacity of HT29 cells in response to platelets (Figure 6B and Supplementary Figure S5A).

To verify whether DG-041 inhibited platelet activation in our coculture model, we assessed its effect on TXB_2_ levels measured in platelet-HT29 cell coculture medium. As shown in Figure 6C, the EP3 antagonist caused a significant reduction in TXB_2_ levels. Similarly, DG-041 profoundly diminished PGE_2_ levels (Figure 6D). The simultaneous inhibition of the two prostanoids by DG-041 may suggest that antagonism of platelet EP3 signaling leads to reduced availability of arachidonic acid (AA).

### Antagonism of the platelet ADP receptor P2Y12 affects EMT and migration in platelet-cancer cell cocultures

To support the hypothesis that the interference with the platelet Gαi signaling inhibits the induction of a mesenchymal-like cancer cell with migratory properties, we tested the effect of a P2Y12 antagonist, ticagrelor. P2Y12 is an important platelet receptor whose activation by ADP leads to several platelet responses, at least in part, via the inhibition of a Gαi-type G protein and reduction in cAMP levels [13].

**Figure 7:**
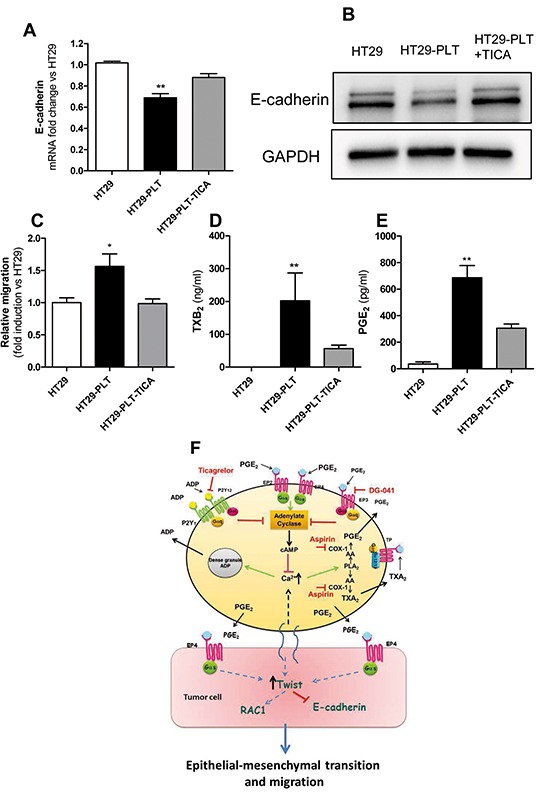
An antagonist of platelet P2Y12 receptor affects EMT and migration of HT29 cells exposed to platelets HT29 cells were cultured alone (1×10^6^) or cocultured with platelets (1×10^8^), in the absence (HT29-PLT) or presence of the P2Y12 antagonist Ticagrelor(10 μM)(HT29-PLT -TICA) for 20h (for gene expression analyses) and 40h (for protein expression analyses). HT29 cells were extensively washed with PBS to remove platelets, harvested with trypsin and the expression levels of E-cadherin mRNA **A.** and protein (normalized to GAPDH) **B.** respectively, were assessed by qPCR and Western blot (one of three representative Western blots is shown). **C.** In the same experimental conditions, the migratory properties of HT29 were assessed using the Boyden chamber assay (as reported in the legend to Figure 2). **D.** and **E.** TXB_2_ and PGE_2_ levels, respectively, were measured in culture media of HT29 cells either cultured alone (HT29), cocultured with platelets (HT29-PLT) in the absence and presence of Ticagrelor (HT29-PLT-TICA) for 40h. **P<0.01, *P<0.05 vs all the other conditions. Data are reported as mean ± SEM (n=3-6). **F.** Platelet-derived PGE_2_ and a direct platelet-tumor cell interaction synergize to promote EMT and migration through the induction of Twist1.Twist1 is involved in the downregulation of E-cadherin and the upregulation of RAC1. These events lead to enhanced migratory capacity of HT29 cells. Platelet-induced EMT and migration are prevented by the inhibition of platelet function by aspirin, an inhibitor of COX-1, ticagrelor, an antagonist of P2Y12 or DG-041, an antagonist of the PGE_2_ receptor EP3.

Ticagrelor prevented the down-regulation of E-cadherin in HT29 cells cocultured with platelets (Figures 7A and 7B) and it inhibited the enhanced migratory capacity of HT29 cells (Figure 7C and Supplementary Figure S5B). Similarly to DG-041, ticagrelor caused the simultaneous inhibition of TXB_2_ and PGE_2_ production (Figure 7D and 7E) thus suggesting an inhibitory effect on the release of AA from platelet membrane phospholipids.

## DISCUSSION

In the present study, we have found that coculturing HT29 cells with human platelets led to the induction of mesenchymal-like cancer cells with enhanced capacity of cell mobility and proaggregatory action on platelets. Our pharmacological studies showed that the inhibition of platelet activation, by aspirin, the EP3 antagonist DG-041 or the P2Y12 antagonist ticagrelor, was effective to prevent these changes. These treatments inhibited direct cancer cell-platelet interactions and PGE_2_ production by platelets, thus preventing the activation of EP4 on HT29 cells (Figure 7F).

Different tumor cell surface molecules have been reported to be involved in the binding to various platelet receptors leading to platelet activation [27]. Dovizio et al., [5] have recently shown that the collagen receptor GPVI is involved in the binding of platelets to HT29 cells via galectin-3, which is expressed in tumor cells and contains a collagen-like domain. Here, we have characterized the contribution of platelet PGE_2_ to the induction of EMT in HT29 cells.

The interaction of platelets with HT29 cells caused the upregulation of Twist1 associated with the downregulation of E-cadherin and induction of RAC1. Twist1 is a transcription factor important in embryonic development, and plays an essential role in tumor metastases [20]. It can transcriptionally repress E-cadherin in breast cancer cells [21]. An inverse correlation between the expression levels of Twist1 and E-cadherin has been observed in human invasive lobular carcinomas [20]. Moreover, Twist1 may be involved in the acquisition of the migratory capacity of HT29 cells (Figure 7F). In fact, it has been shown that Twist1 induces the motile stem-like cancer cell phenotype via the activation of the Twist1-let-7i-NEDD9 axis leading to RAC1 activation [25].

The induction of Twist1 and the downregulation of E-cadherin in HT29 cells by platelets were mediated by the release of PGE_2_. Other signaling pathways triggered by the interaction of platelets with cancer cells further increased the expression of Twist1 and contributed to the formation of a migratory cancer cell presumably through the induction of RAC1 (Figure 7F).

In addition to PGE_2_, another platelet mediator relevant to cancer development is TXA_2_. HT29 cells may be insensitive to an enhanced release of TXA_2_ by activated platelets, as a result of the absence of TP receptor (5 and Supplementary Figure S4). However, TXA_2_ may play a critical role in tumor colonization by facilitating the interaction of platelets with metastatic tumor cells and endothelial cells [28] and for its involvement in angiogenesis [29]. In patients with CRC, the systemic biosynthesis of TXA_2_ was enhanced and the administration of low-dose aspirin inhibited it [30]; these results suggest that platelets are activated in CRC.

Here, we show that the injection of HT29 cells with a mesenchymal-like phenotype, into the circulation of NSG mice, activated platelets which released enhanced levels of TXA_2_ and PGE_2_. The prothrombotic properties of cancer cells undergoing EMT contributed to the development of metastases. In fact, the administration of low-dose aspirin, which inhibited platelet activation and the biosynthesis of prostanoids, was associated with reduced formation of metastases.

In conclusion, low-dose aspirin and possibly other antiplatelet agents may represent effective antimetastatic agents by averting the stem cell mimicry of cancer cells induced by platelets. Mesenchymal-like cancer cells have enhanced capacity to activate platelets, thus promoting the formation of platelet aggregates surrounding tumor cells and this event is central in the development of cancer metastases [6]. Our results provide mechanistic understanding of the reported antimetastatic properties of low-dose aspirin in post-hoc analyses of randomized trials for cardiovascular prevention [7,8], and reinforce the rationale for performing adjuvant trials of low-dose aspirin, and possibly other antiplatelet agents, in CRC patients (http://www.addaspirintrial.org).

## MATERIALS AND METHODS

### *In vitro* experiments: platelet-HT29 cell cocultures

The human colon carcinoma cell line HT29 was obtained from European Collection of Cell Cultures (ECACC, Salisbury, UK). Cells were cultured, as previously described [5]. In all experiments, HT29 cells (1×10^6^) were seeded in six multiwell plates containing 2 ml of McCoy's 5A (Invitrogen, Milan, Italy) supplemented with FBS 0.5% and 1% P/S and polymixyn B sulfate 10 μg/ml. Human washed platelets were isolated from leukocyte concentrates derived from healthy volunteers after obtaining written informed consent (Transfusion Centre, at S.S. Annunziata Hospital, Chieti, Italy), as previously described [5]. Leukocyte contamination was evaluated by fluorescence microscopy using propidium iodide staining and by flow cytometry with CD45 and CD14 antibodies (BD Biosciences, Palo Alto, CA, USA). Leukocyte counts were always less than 1/10,000. In addition, monocyte contamination was evaluated by amplification of CD14 mRNA by RT–PCR. One hundred microliters of platelet suspension (1×10^8^ cells) were added to HT29 cells (1×10^6^) and the incubation continued up to 20-40h. As control condition, HT29 cells were incubated with 100μl of culture medium and cultured alone. In some experiments, platelets were pre-treated for 30min with aspirin 300μM (to completely suppress platelet COX-1 activity) or dimethyl sulfoxide (DMSO, at the concentration of 0.1%); then platelets were washed twice, and co-cultured with HT29 cells both in the absence and in the presence of PGE_2_ (5 nM) for 20h and 40h. In the cocultures of platelets and HT29 cells, we tested the effects of :i) L-161,982, 10 μM, a potent and selective EP4 receptor antagonist [24](Tocris Bioscience, Bristol, UK); ii) DG-041, 3 μM, a direct-acting EP3 antagonist [14] currently being evaluated in Phase II clinical trials (kindly provided by Dr R.M. Breyer, Vanderbilt University Medical Center, Nashville, TN), iii) ticagrelor [12,13],10 μM (Cayman Chemical, Ann Arbor, MI), a cyclopentyl-triazolopyrimidine orally active, reversible, and selective antagonist of platelet P2Y12, clinically used in the prevention of cardiovascular events. The chemical structures of EP antagonists used are reported in Supplementary Material and Methods.

At the end of the incubation (40h), conditioned media were harvested from HT29 cells and centrifuged at 700g for 10min to discard cell debris, and supernatants were analyzed for the levels of TXB_2_ (the stable hydrolysis product of TXA_2_) and PGE_2_ by previously described immunoassay techniques [5]. HT29 cells were extensively washed with PBS (Sigma-Aldrich) to remove platelets (as shown in Supplementary Figure 1, HT29 cells were substantially devoid of platelets) and harvested with trypsin (Invitrogen) and assayed for protein expression (i.e. E-Cadherin and Twist1) by Western blot technique as previously described [5] and briefly reported in Supplementary Materials and Methods. In the 20h-cocultures after extensive washing of HT29 cells, total RNA was isolated, as previously described [5], and mRNA levels of E-Cadherin, Twist1 and RAC1 were evaluated by quantitative PCR (qPCR) (ref[5] and Supplementary Materials and Methods) In some experiments, the migratory capacity of HT29 cells was evaluated both *in vitro* (by Boyden chamber migration assay) and *in vivo* (in a mouse model of hematogenous metastases) as described below.

In another set of experiments, HT29 cells were cultured alone in the absence or in the presence of exogenous PGE_2_ (Cayman Chemicals; 5 nM) or with vehicle (DMSO) for 20 or 40h for the assessment of the migratory capacity *in vitro*, the expression of Twist1 and E-cadherin mRNA. Under this experimental conditions, we tested the effects of: i) SC-51322, a potent EP1 prostanoid receptor antagonist [22]; ii) PF 04418948, an EP2 antagonist [23]; and iii) L-161,982, an EP4 receptor antagonist [24](Tocris Bioscience, Bristol, UK).

### Boyden chamber migration assay

Cell migration assays were performed using a Boyden chamber consisting of transwell inserts (8 μm pore polycarbonate membrane; Corning, NY, NY) mounted on 24-well multiplates. Briefly, HT29 cells were cultured alone or with platelets for 40h as described above. After the incubation, HT29 cells were detached, counted and seeded (1 x10^5^ cells per insert) onto the upper chamber of transwell filters in 24-well multiplates. Cells were allowed to migrate for 40h, at 37°C in 5% CO_2_. Non-migrated cells were carefully removed with a cotton swap, while cells migrated to the lower surface of the filters were fixed in paraformaldehyde (4% in PBS) and stained with a solution of 0.5% crystal violet (Sigma-Aldrich) - 50% methanol. Migrated HT29 cells were then quantified under a microscope at 40x magnification. Fold induction of migrated cells vs control (i.e. untreated HT29 cells) was evaluated.

### Tumor cell-induced platelet aggregation (TCIPA)

Fresh whole blood samples were collected from healthy volunteers, after obtaining written informed consent, and anticoagulated with a 1/6 volume of acid-citrate dextrose (ACD, Baxter, Florence, Italy). Platelet-rich plasma (PRP) was obtained by blood centrifugation at 200g for 10min without brake. Platelets were sedimented by centrifugating PRP at 700g for 15min without brake and then resuspended in Hepes buffer pH 7.4 (10mM Hepes, 145mMNaCl, 5mMKCl, 0.5mM Na_2_HPO_4_and 6mM glucose) at a concentration of 2×10^8^/ml.

The effect of HT29 cells on platelets was studied by light aggregometer (Chrono-Log, Havertown, PA). Briefly, 200 μl of platelets (0.4×10^8^ cells) were placed in the aggregometer and incubated for 2min at 37°C after the addition of 1mM MgSO_4_ and 1mM CaCl_2_. After the incubation, platelets were subjected to stirring at 900rpm prior to the addition of tumor cells. TCIPA was initiated by the addition of 50μl of tumor cells (HT29 cells cultured alone or cocultured with platelets for 40h *in vitro*), resuspended in Hepes buffer at the final concentration of 0.8×10^7^/ml, and the reaction was monitored and analyzed using the Aggro-link data processing system (Chrono-Log) for up to 20min. Platelet aggregation was expressed as a percentage of the maximum aggregation rate.

### *in vivo* mouse model of hematogenous metastasis of human HT29 cells

NSG mice [31] were purchased from Jackson Laboratories (Bar Harbor, Maine). The animals were housed in cages up to five mice each and acclimated for 1 week under conditions of controlled temperature (20 ± 2°C), humidity (55 ±10%), and lighting (7:00 a.m. to 7:00 p.m.). For all the experiments, mice were housed under specific pathogen-free conditions and allowed free access to food and water. Age-matched males (7-9 weeks old, weighing 25-30g) were used for all experiments. Experiments were performed in accordance with local laws and the Council of the European Communities Directive of November 24, 1986 (86/609/EEC) guidelines for the care and use of laboratory animals. The study was approved by the Institutional Animal Use and Care Committee (“G. d'Annunzio” University, Chieti, Italy) (protocol no. 62/2011).

All efforts were made to minimize animal suffering and to reduce the number of animals used.

HT29 cells, cultured alone or with platelets for 40h (as described above), were detached and washed twice to remove platelets (as shown in Supplementary Figure S1, HT29 cells were substantially devoid of any platelets), and resuspended in Hank's BSS medium (PAA Laboratories GmbH) at a concentration of 5×10^6^ cells/ml. Two-hundred μl of this cell suspension (corresponding to 1×10^6^ cells) were injected intravenously (i.v.) into the lateral tail vein of NSG mice (n=20). The mice were subdivided into 4 groups: (1) 5 mice were injected with Hank's BSS medium without cancer cells (baseline condition); (2) 5 mice were injected with HT29 cells cultured alone; (3) 5 mice were injected with HT29 cells previously incubated *in vitro* with platelets; (4) 5 mice were treated with oral aspirin (20 mg/kg daily) before and after the injection of HT29 cells that were previously incubated *in vitro* with platelets. Aspirin (Sigma Aldrich, Milan Italy) was dissolved in water (2mg/ml) and administered daily by oral gavage at the dose of 20 mg/kg/mouse [corresponding to the dose of 150 mg daily for humans, using the body surface area (BSA) normalization method] [18]. Aspirin was administered once a day starting from 4 days before the injection of HT29 cells co-cultured with platelets, until a week after the injection.

In all groups of mice, a retro-orbital blood sample was taken from the contralateral eye of each mouse for the measurement of serum TXB_2_ [19] (after whole blood clotting for 1h at 37°C), and urine samples were collected with the use of metabolic cages over a 24-hour period, for the assessment of urinary levels of the major enzymatic metabolites of TXA_2_, PGI_2_ and PGE_2_ [32].

One week after the injection, mice were sacrificed, lungs were collected, formalin-fixed and submitted for histopathology and the hematoxylin-eosin-stained microscopic sections were scored for the presence of pathologic lesions.

In additional experiments, mice (5 for each group) were injected with HT29 cells cultured alone or HT29 cells co-cultured with platelets not exposed or exposed to aspirin *in vitro* (as reported above) and urinary samples were collected to assess the levels of the enzymatic metabolites of TXA_2_ and PGE_2_.

### Metastatic score

Formalin-fixed lungs were submitted for histopathology and the hematoxylin-eosin-stained microscopic sections were scored for the presence of pathological lesions. Pulmonary micrometastases were scored by the size (S) of the majority of lesions as: (1)small lesions containing approximately 25-100 tumor cells; (2)medium-sized lesions containing approximately 100-500 tumor cells; (3)large lesions containing more than 500 tumor cells. Lung involvement was also scored in term of the surface area (A) interested by lesions: 0, no visible metastatic lesions; 1, < 5% of the lung surface involved; 2, between 5% and 50% of the lung surface involved; 3, >50% of the lung surface involved. A metastatic score was obtained by combining the two partial scores as size × involved area (S × A).

### Mass spectrometric analysis of urinary prostanoid metabolites

Urine samples (at least 300 μl/mouse) were collected and immediately frozen. Systemic production of PGI_2_, PGE_2_, and TXA_2_ was determined by quantifying their major urinary metabolites: 2,3-dinor 6-keto-PGF_1_α (PGI-M); 7-hydroxy-5,11-diketotetranorprostane-1,16-dioic acid (PGE-M); and 2,3-dinor TXB_2_ (TX-M), respectively, by mass spectrometry as described previously [32]. Data were normalized for urinary creatinine (Oxford Biomedical Research).

### Statistical analysis

All values are reported as mean ± SEM. Statistical analysis was performed using GraphPad Prism Software (version 5.00 for Windows; GraphPad, San Diego, CA). Briefly, Student's t test was used to compare the means of two independent groups to each other, whereas one-way analysis of variance followed by Newman-Keuls post-test was used to compare the means of more than two independent groups. P values < 0.05 were considered statistically significant.

## SUPPLEMENTARY MATERIALS AND METHODS FIGURES


