# Supplementary material for: Aspirin prevents colorectal cancer metastasis in mice by splitting the crosstalk between platelets and tumor cells
Source: Oncotarget. 2016 Apr 8;7(22):32462–77. doi: 10.18632/oncotarget.8655 (PMC5078026; doi:10.18632/oncotarget.8655)
Supplement: Supplementary file 1 [file oncotarget-07-32462-s001.pdf]

Aspirin prevents colorectal cancer metastasis in mice by splitting the crosstalk between platelets and tumor cells

SUPPLEMENTARY MATERIALS AND METHODS

Chemical structure of EP antagonists

SC-51322, (8-Chloro-2-[3-[(2-furanylmethyl)thio]-1-oxopropyl]-dibenz(Z)[b,f][1,4]oxazepine-10(11H)-carboxylic acid hydrazide), a potent EP1 prostanoid receptor antagonist; PF 04418948, 1-(4-Fluorobenzoyl)-3-[[(6-methoxy-2-naphthalenyl)oxy]methyl]-3-azetidinecarboxylic acid, an EP2 antagonist; L-161,982 (N-[[4'-[[3-butyl-1,5-dihydro-5-oxo-1-[2-(trifluoromethyl)phenyl]-4H-1,2,4-triazol-4-yl]methyl][1,1'-biphenyl]-2-yl]sulfonyl]-3-methyl-2-thiophenecarboxamide), an EP4 receptor antagonist.

Western blot analysis

Protein analysis was carried out in lysates of HT29 cells cultured alone or cocultured with platelets for 40h by Western blot. Protein expression was detected using the specific antibodies: E-cadherin (Santa Cruz Biotechnology, sc-7870, dilution 1:1000), Twist1 (Sigma-Aldrich, T6451, dilution 1:1000). GAPDH (Santa Cruz Biotechnology, sc-20357, dilution 1:1000) was used as loading control. Briefly, cells were lysed in PBS containing 1% TritonX-100, phenylmethylsulfonyl fluoride (PMSF; 1 mM) and protease inhibitors (Thermo Scientific, Waltham, MA, USA), and the protein concentration, in each sample, was assessed by Bradford assay (Bio-Rad). Cell lysates were subjected to Western blot analysis using conventional SDS-PAGE gel electrophoresis and protein transfer to PVDF filters (Bio-Rad). Membranes were incubated with the indicated antibodies and developed using Western Lightning Plus-ECL (Perkin Elmer, Waltham, MA, USA). Results were obtained using a digital imaging system Alliance 4.7 (UVITEC, Cambridge, UK).

mRNA analysis

Total RNA was isolated from HT29 cells after 20h treatment using Pure link RNA Mini kit (Life Technologies) to assess mRNA expression levels of TWIST1, E-cadherin and RAC1 and normalized with GAPDH levels using a 7900HT Real-Time PCR system (Applied Biosystems, Foster City, CA). Two μg of extracted RNA were retro-transcribed using iScript cDNA synthesis kit (BioRad, Hercules, CA, USA). One hundred ng of cDNA was used for the reaction mixture and the amplification of TWIST1, E-cadherin and GAPDH levels was performed using TaqMan gene expression assays (Hs01675818_s1, Hs01023894_m1, and Hs-02758991, respectively) (Applied Biosystems) according to the manufacturer's instructions. For RAC1, 100ng of cDNA was used for the reaction mixture, and the amplification of RAC1 and GAPDH was performed using iTAQ Universal SYBR Green Supermix (Bio-Rad) and these couples of primers: Rac 1-fwd: 5′-AGTGGTATCCTGAGGTGCG-3′; rev: 5′-TAGACCCTGCGGATAGGTGA-3′; GAPDH-fwd: 5′-TCACCAGGGCTGCTTTTAAC-3′; rev:5′- GACAAGCTTCCCGTTCTCAG-3′.

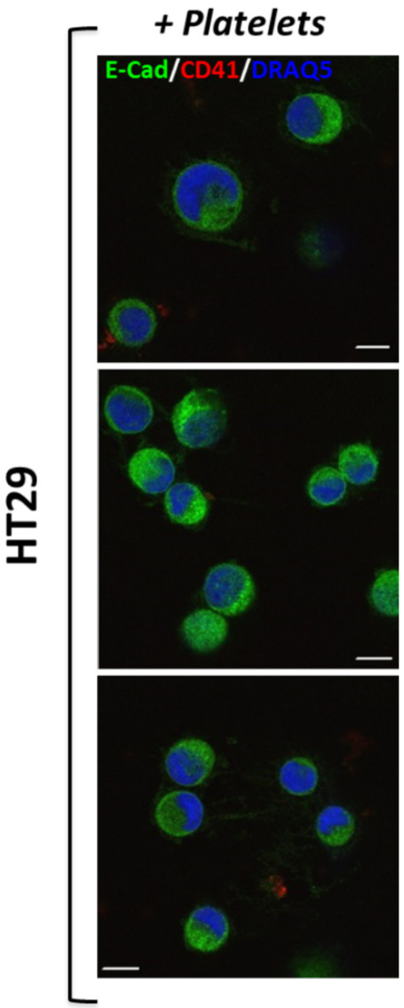

Supplementary Figure S1: Immunofluorescence staining for platelets CD41 (red) and HT29 cell E-Cadherin (green). HT29 (1×10^6 cells) were co-cultured with platelets (1×10^8) for 40h; then, cells were washed extensively with PBS pH **7**.4, resuspended in Hank's BSS medium and tumor cells were assessed for the presence of platelets by confocal microscopy. Briefly, HT29 cells were incubated overnight at 4°C with polyclonal antibodies anti-E-Cadherin (1:100, Santa Cruz Biotechnology, Dallas, TX) and anti-CD41 (1:100, Santa Cruz Biotechnology). Cells were then washed three times with PBS-0.1% tween and incubated with secondary antibodies goat anti-rabbit Alexa Fluor 488 and goat anti-mouse Alexa Fluor 546 (1:1000, Life Technologies, Monza Italy) for 1h at room temperature. Cells were washed three times with PBS-0.1% tween and incubated with DRAQ5 as a nuclear marker (1:1000, Thermo Scientific, Waltham, MA, USA) for 5min. Finally, cells were washed and mounted in slides with Diamond antifade mounting. Nuclei are stained in blue (DRAQ5). Three representative images of cells are shown. Scale bar=10µm.

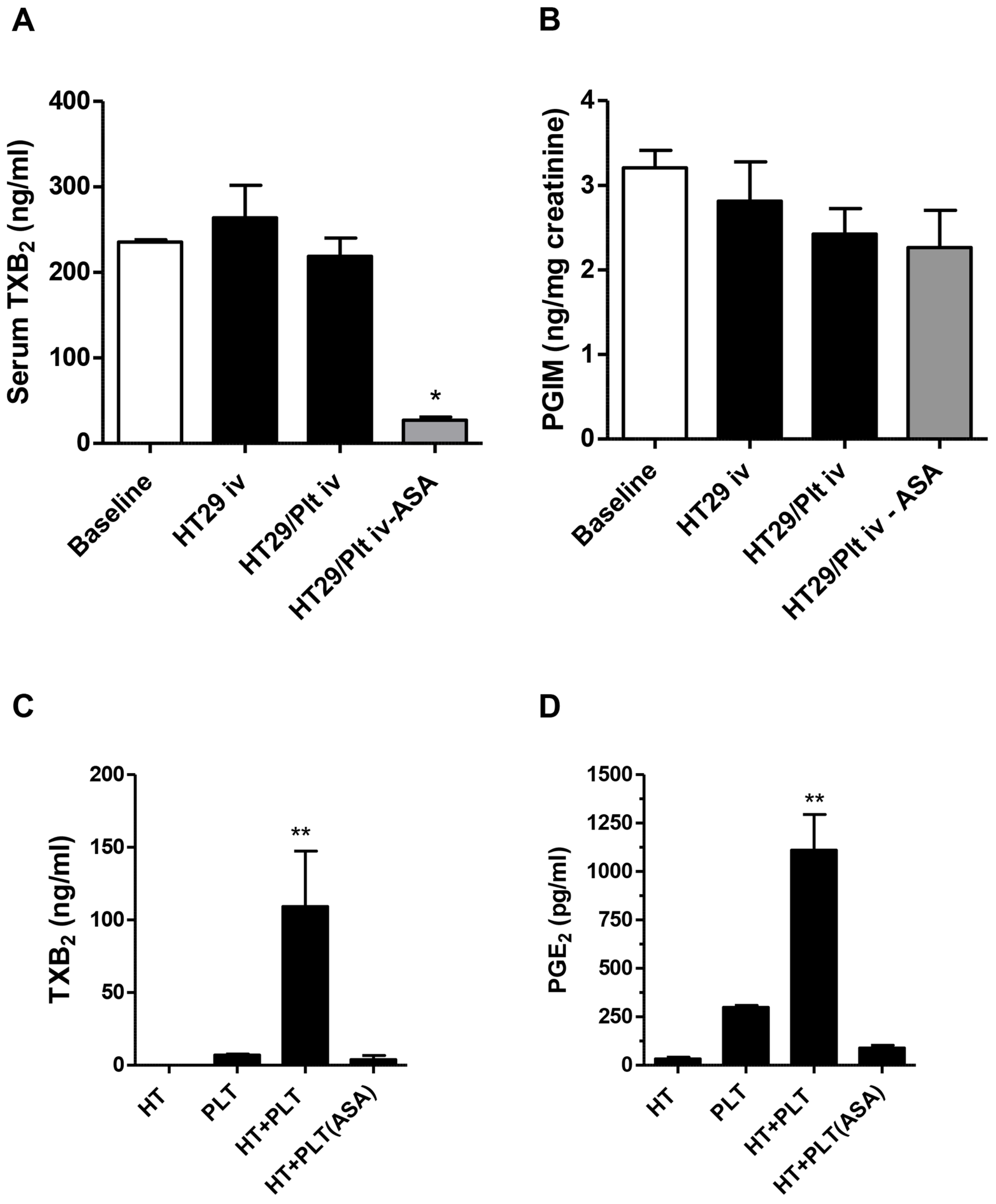

Supplementary Figure S2: **A.** and **B.** HT29 cells (1×10^6) cultured alone (HT29) or co-cultured with platelets (HT29-PLT) for 40h, were injected into the lateral tail vein of NSG mice (n=5 each group). In HT29-PLT-ASA group (n=5), mice were treated with aspirin (20 mg/kg, p.o. daily) before and up to 7 days after the injection of the cells; (A) in all groups of mice, a retro-orbital bleed sample was taken from the contro-lateral eye of each mouse for the measurement of serum TXB_2 production in whole blood allowed to clot for 1h at 37°C, *P<0.05 vs all other conditions; (B) 24h urine samples were collected to assess the urinary excretion of 2,3-dinor-6-keto-PGF1α (PGI-M). Values are reported as mean ± SEM (n=5); **C.** and **D.** levels of TXB_2 and PGE_2 detected in conditioned medium of platelet-HT29 cell cocultures, respectively; values are reported as mean ± SEM (n=4-5) **P<0.01 vs all the other conditions.

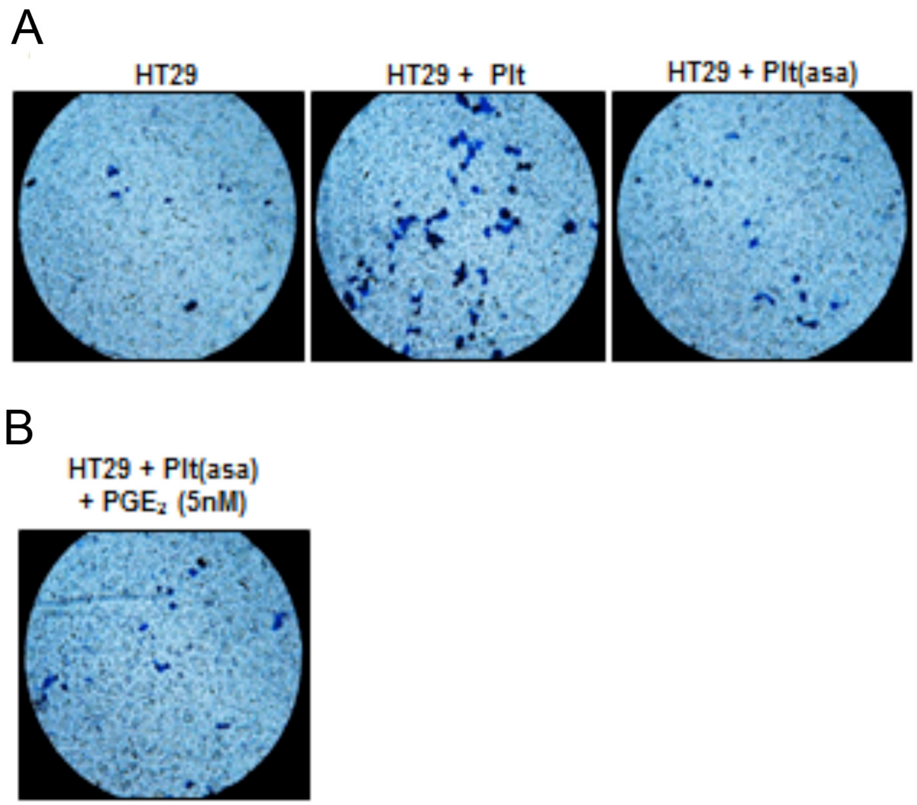

Supplementary Figure S3: Cell migration assay performed using a Boyden chamber in different experimental conditions. **A.** HT29 cells (1×10^6 cells) were co-cultured for 40h with human platelets (1×10^8 cells) untreated or treated with aspirin; **B.** HT29 cells were co-cultured with aspirinated platelets in the presence of exogenous PGE_2 (5nM). After the incubation, HT29 cells were detached, counted and seeded (1×10^5 cells per insert) onto the upper chamber of transwell filters in 24-well multiplates; cells were allowed to migrate for 40h, at 37°C in 5% CO_2. Non-migrated cells were carefully removed with a cotton swap, while cells migrated to the lower surface of the filters were fixed in paraformaldehyde (4% in PBS) and stained with a solution of 0.5% crystal violet (Sigma-Aldrich) - 50% methanol. Migrated HT29 cells were then quantified under a microscope at 40x magnification.

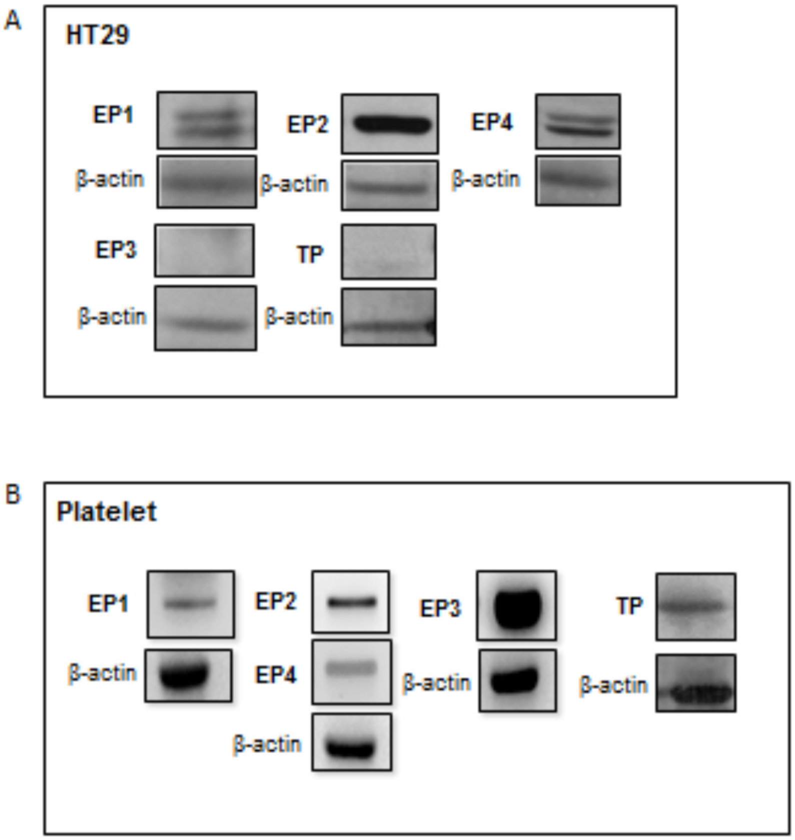

Supplementary Figure S4: HT29 cells A. and washed platelets B. were analysed by Western blot. 15 µg of proteins were analyzed by SDS polyacrylamide gel electrophoresis (SDS-PAGE). A separating gel of 8-9% was used for EP1, EP2, EP3 and EP4 and TP. The resolved proteins were transferred electrophoretically to polyvinylidene difluoride (PVDF) membrane. The membrane was saturated with a solution of 5% non-fat milk in tris-buffered saline-0.1% Tween-20 (TBS-Tween-20), followed by the incubation with primary antibodies diluted in TBS-Tween-20: anti-EP1, EP2, EP3, EP4 and TP polyclonal antibodies (1:1000, Cayman Chemical, Ann Arbor, MI) and anti-β-actin polyclonal antibodies (1:1000, Santa Cruz, USA). All primary antibodies were incubated in TBS-Tween-20 overnight at 4°C; then, membranes were washed with a solution of 5% non-fat milk TBS-Tween-20 for 10min and twice with TBS-Tween-20 for 10min and incubated with secondary antibodies for 1h at room temperature: donkey anti-goat IgG-horseradish peroxidase (HRP) conjugate (1:2000, Santa Cruz) was used for β-actin; anti-rabbit IgG-HRP conjugate (1:2000, Sigma Aldrich, Milan Italy) was used for EP1, EP2, EP3 and EP4 and TP. The acquisition of specific bands was performed using Alliance 4.7 (UVITEC, Cambridge, UK) and the quantification of optical density (OD) of different specific bands was analyzed usingAlliance 1 D software (UVITEC, Cambridge, UK) and normalized to the OD of β-actin.

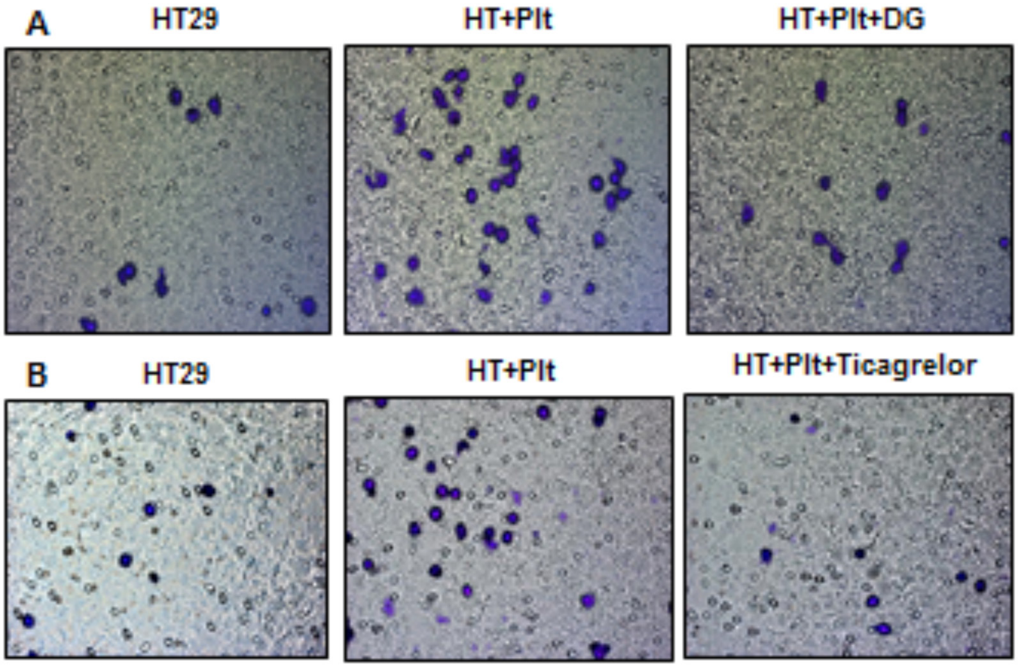

Supplementary Figure S5: Migration assays were carried out in Boyden chambers as described above. Images show representative photographs of 40x fields of transwell inserts from migration assays using either HT29 cells cultured alone (HT29), HT29 cells cocultured with platelets (HT+Plt) or HT29 cells cocultured with platelets using the indicated drugs: **A.** DG-041; **B.** Ticagrelor.
